# Effects of NMDA-receptor blockade by ketamine on mentalizing and its neural correlates in humans: a randomized control trial

**DOI:** 10.1038/s41598-023-44443-6

**Published:** 2023-10-11

**Authors:** Sven Wasserthal, Mirko Lehmann, Claudia Neumann, Achilles Delis, Alexandra Philipsen, René Hurlemann, Ulrich Ettinger, Johannes Schultz

**Affiliations:** 1 Division of Medical Psychology, Department of Psychiatry and Psychotherapy, University Hospital of Bonn, Venusberg-Campus 1, 53127 Bonn, Germany; 2 Department of Psychology, University of Bonn, Bonn, Germany; 3 Department of Anaesthesiology and Intensive Care Medicine, University Hospital Bonn, Bonn, Germany; 4 Department of Psychiatry and Psychotherapy, University Hospital of Bonn, Bonn, Germany; 5 Department of Psychiatry, School of Medicine and Health Sciences, University of Oldenburg, Oldenburg, Germany; 6 Center for Economics and Neuroscience, University of Bonn, Bonn, Germany; 7 Institute for Experimental Epileptology and Cognition Research, Medical Faculty, University of Bonn, Bonn, Germany

**Keywords:** Neuroscience, Social neuroscience

## Abstract

Schizophrenia is associated with various deficits in social cognition that remain relatively unaltered by antipsychotic treatment. While faulty glutamate signaling has been associated with general cognitive deficits as well as negative symptoms of schizophrenia, no direct link between manipulation of glutamate signaling and deficits in mentalizing has been demonstrated thus far. Here, we experimentally investigated whether ketamine, an uncompetitive N-methyl-D-aspartate receptor antagonist known to induce psychotomimetic effects, influences mentalizing and its neural correlates. In a randomized, placebo-controlled between-subjects experiment, we intravenously administered ketamine or placebo to healthy participants performing a video-based social cognition task during functional magnetic resonance imaging. Psychotomimetic effects of ketamine were assessed using the Positive and Negative Syndrome Scale. Compared to placebo, ketamine led to significantly more psychotic symptoms and reduced mentalizing performance (more “no mentalizing” errors). Ketamine also influenced blood oxygen level dependent (BOLD) response during mentalizing compared to placebo. Specifically, ketamine increased BOLD in right posterior superior temporal sulcus (pSTS) and increased connectivity between pSTS and anterior precuneus. These increases may reflect a dysfunctional shift of attention induced by ketamine that leads to mentalizing deficits. Our findings show that a psychotomimetic dose of ketamine impairs mentalizing and influences its neural correlates, a result compatible with the notion that deficient glutamate signaling may contribute to deficits in mentalizing in schizophrenia. The results also support efforts to seek novel psychopharmacological treatments for psychosis and schizophrenia targeting glutamatergic transmission.

## Introduction

People with schizophrenia frequently show deficits in social cognition, a cognitive domain comprising multiple processes including social cue perception, mentalizing, regulation of emotion and experience sharing^1,2^. These impairments affect community functioning more than impairments in other cognitive domains^3,4^. Deficits in mentalizing—the ability to infer mental states in others or oneself—can be observed across all stages of the disorder^5–7^ and can consist of over- or undermentalizing, corresponding to excessive or insufficient attribution of mental states to other agents^8^. While overmentalizing is more frequently associated with positive symptoms—paranoid thoughts often consist of attributing more intentions to social situations than are actually present—undermentalizing has been associated with negative or disorganized symptoms that numb experiences of the surrounding world, leading to diminished attribution of intentions to others^8–10^. Neural correlates of mentalizing deficits in schizophrenia include abnormal activation of the superior temporal sulcus (STS), anterior cingulate cortex (AC), bilateral temporo-parietal junctions (TPJ), medial prefrontal cortex (mPFC) and precuneus^11–14^. Both hypo- and hyperactivation of STS and precuneus have been reported during mentalizing^15,16^.

While in some instances social cognition or mentalizing were shown to improve upon treatment with atypical antipsychotics^17,18^, deficits in these domains remain largely unchanged during the course of the illness^19^. At present, evidence about the use of antipsychotics targeting faulty dopamine-signaling via D2-receptor-binding to treat social cognition deficits appears inconclusive^20^. However, various studies point to a significant role of the glutamatergic *N*-methyl-D-aspartate (NMDA) receptor in the development of negative and cognitive symptoms. The mechanism by which NMDA-receptors are believed to contribute to the provenance of cognitive symptoms involves hypofunctioning of these receptors on gamma-aminobutyric acid (GABA) interneurons. In this hypothesis, impairment of these interneurons then leads to disinhibition of pyramidal cells in corticolimbic circuits. To test theories about deficient glutamate signaling and its connection to schizophrenia in a behavioural design, multiple studies have used NMDA-receptor antagonists to evoke psychosis-like states in healthy subjects or animals^21^. One such uncompetitive NMDA-receptor antagonist is ketamine. Mainly used to induce a narcotic state, it blocks the NMDA-receptor ion channels and thus the influx of ${\mathrm{Ca}}^{2+}$, partly explaining hypofunction on GABAergic interneurons and leading to locally specific hyper- or hypoactivity in the brain^22^.

Since discovery of its psychotomimetic qualities, subanesthetic ketamine has been used as a model for psychosis in humans and rodents^23^. In a study of Phensy and colleagues, male mice postnatally injected with ketamine showed disrupted social investigation patterns when introduced to unfamiliar conspecifics^24^. Disruption of these patterns has repeatedly been shown in similar experiments with ketamine in the animal model^25,26^. However, mentalizing, a key social cognitive skill deficient in schizophrenia, is arguably best tested in humans. And while studies with human subjects have explored ketamine effects with respect to memory^27,28^, modulation of emotion-cognition interaction^29^, response inhibition^30^, probabilistic inference^31^, metacognition^32^ and processing of emotional faces^33^, to our knowledge, no study has investigated the influence of ketamine on mentalizing.

The aim of this study was to find out whether manipulating NMDA-receptor activation via ketamine would influence mentalizing and its neural correlates in healthy volunteers. We used a modified version of the Movies for the Assessment of Social Cognition (MASC) task by Dziobek and colleagues to assess the level of mentalizing (normal, over-, under- or no mentalizing)^34^, a task on which patients with schizophrenia were shown to exhibit different mentalizing patterns than controls^8,34,35^. We expected volunteers subjected to ketamine to display more undermentalizing or no-mentalizing than individuals receiving placebo. We also predicted abnormal activation of brain regions associated with mentalizing.

## Methods

### Participants

387 participants were recruited via online message boards at the University of Bonn and screened for eligibility via an online questionnaire between June 2019 and September 2020. Among 85 eligible participants, 70 participants (mean age = 24.18, SD = 4.17, range = 18–34, 37 female) took part in the MRI study. Randomization algorithms were created by U.E. and all personnel except anesthesiologists remained blinded until preprocessing of fMRI data had been conducted. There were no significant differences between ketamine and placebo groups in terms of gender [*X*^*2*^ (1, N = 63) = 0.15, *p* = 0.701] or age [t(61) = 0.80, p = 0.427; independent samples t-test].

If participants showed a dominance for the right hand^36^, were non-smoking^37^, considered themselves non-claustrophobic and had never taken or received ketamine, they were invited for an on-site screening. The on-site screening consisted of a short structured clinical interview (Mini-International Neuropsychiatric Interview v. 5.0), items assessing positive symptom load indicative of psychosis risk from the Structured Interview for Prodromal Symptoms (SIPS)^38^, a urine drug-screen (SureStep Urine Multi Drug, Innovacon Inc.) and—for female participants—a pregnancy test (hCG cassette, Alere). Details on inclusion criteria can be found in Table 1. One participant cancelled an appointment for the MRI experiment after randomization and will thus be treated as dropout.

**Table 1:** List of exclusion criteria with instruments.

Exclusion criterion	Instrument
Tobacco use	Fagerström test for nicotine dependence
Left-handedness	Edinburgh Inventory of Handedness
Current or past psychiatric diagnosis	MINI v5 (excluding “K”-scale, only filter-items)
Clinical high risk for psychosis	SIPS items P1-P5, cutoff value ≥ 3
Current (or past) drug abuse	Urine drug screening (MINI)
Current pregnancy	Urine pregnancy test
Other relevant medical conditions	Unstructured Interview
No concomitant medication	Unstructured Interview

*MINI* mini-international neuropsychiatric interview, *SIPS* structured interview for prodromal symptoms.

### Ethical approval

The randomized and placebo-controlled study was performed in accordance with the declaration of Helsinki after being approved by the local ethics committee at the Department of Psychology at the University of Bonn, Germany. Data was collected in an MRI facility at the University Hospital of Bonn between June 2019 and September 2020.

### General procedure

On the day of the experiment, participants were required to arrive with an empty stomach, having fasted food at least 6 h and clear fluids 2 h prior to examination. Participants gave written informed consent and received written instructions. After medical examination by an anesthesiologist, an intravenous access was applied to one arm and participants were led into the MRI scanner. After participants were placed inside the MRI scanner and preparations were complete, the infusion was initialized. The anesthesiologist ensured there were no adverse events and participants were able to familiarize themselves with the substance prior to scanning to avoid anxiety-related adverse effects. The experimental task and MRI data acquisition were then initiated under continuous heart rate and oxygen level monitoring throughout the infusion. After completion of MRI data acquisition, the infusion was stopped, and participants completed the Positive and Negative Syndrome Scale (PANSS) interview with a trained blinded rater.

### Drug administration

A subanesthetic dose of ketamine was delivered via a Graseby 3500 intravenous infusion pump controlled by the STANPUMP software (Steven Shafer, M.D., Anesthesiology Service, PAVAMC 3801 Miranda Ave., Palo Alto, USA). Target plasma levels were 100 ng/ml with an initial bolus administered as a 2 mg/ml solution. The plasma level of 100ng/ml was chosen in accordance with a study of Krystal et al.^23^, who showed that a dose of 0.5 mg/kg (≈ 100–250 ng/ml) ketamine reliably evoked psychotomimetic symptoms. As earlier work has shown that blood-plasma-levels using this equipment were close to the targeted plasma-levels, no blood samples were drawn^30^. A saline solution (0.9% sodium chloride) was used as placebo. The ketamine/saline solution was prepared by an unblinded anesthesiologist. Thirty-four participants received an infusion with racemic ketamine (Ketamin Inresa, 50mg/ml, 10ml solution). Adverse effects were observed in two subjects, who suffered from nausea and low blood pressure. Both subjects were treated accordingly, released home after monitoring, and checked on the day after the experiment by telephone—no subsequent symptoms were reported. Thirty-five participants received a placebo saline infusion using the same setup; none reported adverse effects.

### Experimental task

**Figure 1:**
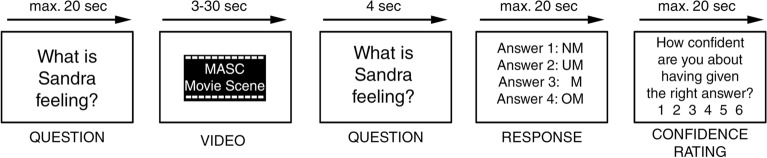
Depiction of one trial of the experimental task, a modified version of the MASC experiment by Dziobek et al.^34^. First, participants were presented with a question about the depicted social situation or the physical surroundings in which the agents interact. Next, a short video clip (max. length = 30s, average = 10s) was presented in which two to four agents interact socially. Afterwards, the question was shown again for 4000 ms and participants were instructed to think about their response. Next, they selected the best fitting response via a button press. Lastly, participants indicated their confidence in the given response. Stimuli are not to scale abbreviations: *NM* no mentalizing, *UM* undermentalizing, *M* mentalizing, *MASC* movie for the assessment of social cognition, *OM* overmentalizing.

We employed a modified version of the MASC task, which assesses mentalizing performance using a set of videos depicting subsequent stages of social interactions between four human agents. The four agents spend an evening together, take part in various activities and interact in friendly, hostile, or romantic ways. The movie is interrupted at key timepoints by four-alternative-forced-choice questions about the currently ongoing social situation (e.g., “what is Michael feeling?”). The four response options represent four possible levels of mentalizing: overmentalizing, “normal” mentalizing, undermentalizing or no mentalizing. The “normal” mentalizing response is the answer that most healthy participants would give and is considered the “correct” answer to the question. Our version of the MASC task was structured as follows (Fig. 1): First, the question to be answered was shown and a button press by the participant initiated the presentation of the video (question phase); then the video sequence was shown (video phase); after the video, the question was presented again for four seconds and participants were instructed to think of an answer during that time. Subsequently, the four possible answers were presented until participants selected their preferred answer (response phase). Finally, they indicated their confidence in their response using a 6-point Likert scale (confidence rating phase; confidence ratings will not be further considered in the present report). To allow contrasting of the neural activation evoked during mentalizing, participants answered an equal number of non-social control questions pertaining to the physical surrounding of the actors. The adaption of the MASC for this study is described in detail in the supplementary materials S1.

### PANSS

The Positive and Negative Syndrome Scale (PANSS) is a semi-standardized medical interview used for measuring symptom severity in schizophrenia. Symptoms are by default subdivided into positive symptoms, negative symptoms and general symptoms^39^. While the scale for positive and negative symptoms are assessed by seven Items each, the general scale features 16 items, each with a scale ranging from 1 to 7. In this study we used the five-factor structure proposed by Lehoux and colleagues to model the effects of ketamine in more detail (positive, cognitive/disorganization, hostility, negative, depression/anxiety)^40^. The PANSS has previously been used in studies analyzing the psychotomimetic effects of ketamine, showing that symptoms of most subscales were significantly increased when participants had received a dose of ketamine^41,42^. PANSS data were collected by a trained and blinded rater not involved in preparation and conduction of the MRI experiment. PANSS data is only available for n = 58 subjects, as the interviewer was absent on one occasion.

### fMRI image acquisition and data analysis

Images were acquired using a 3T Siemens TrioTim MRI scanner (Siemens, Erlangen, Germany). Because participants wore pneumatic headphones, we used a 12- instead of a 32-channel headcoil as the latter was too small to fit participants’ heads with headphones. Using a 12-channel headcoil does not significantly decrease blood-oxygen-level-dependent (BOLD) signal in cortical areas^43^.

Functional images were acquired using a T2*-weighted echo-planar imaging sequence (echo time (TE) = 30 ms, repetition time (TR) = 2500 ms, flip angle = 90°, voxel-size = 2 × 2 × 3 mm, slice thickness = 3mm, field of view (FOV) = 192 mm, 37 slices). For purposes of normalization, a T1-weighted structural scan was acquired (TE = 2.54 ms, TR = 1660 ms, inversion time = 800 ms, slice thickness = 0.8 mm, matrix size = 320 × 320, FOV = 256 mm, flip angle = 9°, voxel-size = 0.8 × 0.8 × 0.8 mm, 208 sagittal slices).

First, fMRI images were checked manually for abnormalities upon which three participants had to be excluded due to artifacts in functional images. Functional EPI images were analyzed using SPM12 (Wellcome Center for Neuroimaging, London, UK, https://www.fil.ion.ucl.ac.uk/spm/software/spm12/) running in MATLAB 2019a (The Math Works, Natick, USA). Head motion was evaluated for every participant using the ART toolbox (https://www.nitrc.org/projects/artifact_detect). Any participant showing a shift in the z-dimension greater than 3mm or exceeding a volume-to-volume movement threshold greater than 1.5mm in 20% of the scans was excluded^44^. Following this procedure, two additional participants were excluded, leaving a final sample of 64 participants. The first six images of every participant were excluded as dummy scans to account for T1 equilibration. Functional images were realigned to the first image in that series using rigid-body transformation. These images were then coregistered to T1 structural scans and normalized to a standard space EPI template volume of the Montreal Neurological Institute (MNI) as provided with SPM12. Normalization failed with one participant, who had to be excluded from further analysis. In a final preparation step, images were smoothed with a Gaussian kernel of 6 mm.

#### fMRI data analysis

For each participant, a fixed-effects general linear model (GLM) was fitted to the preprocessed BOLD signal data using SPM12. The GLM contained separate sets of four regressors for the social and physical trials, modeling the following trial parts: presentation of the question before and after the video, presentation of the video, response, and confidence rating. The model further included six movement regressors representing estimates of rotation and translation created during the realignment step of data preprocessing, and a constant term. While the task was not designed to separate neural activation evoked during the subsequent trial parts, modeling these trial parts separately increased the flexibility of the GLM at small costs on degrees of freedom. The response phase was modelled from offset of the second question presentation to participants’ answer to the mentalizing question, and the confidence rating phase spanned the time between answer to the mentalizing question and locking of the confidence rating. Regression coefficients (parameter estimates) for these regressors were estimated for each voxel of each participant’s brain. Linear contrasts were applied to the individual parameter estimates of the response to the experimental conditions in order to contrast each of these four trial parts of the social and physical trials.

To determine the effect of ketamine on BOLD signal during mentalizing, we first verified that the social cognition task evoked activation in brain regions known to be involved in social cognition under placebo. To this end, we constructed a second-level (random effects) model in SPM that allowed us to contrast neural correlates of social vs non-social cognition under placebo and ketamine in the four trial parts (see Fig. 1). In each participant’s fixed-effects GLM (see above), we calculated the social(SOC) > physical (PHY) contrast for each trial part and imported those contrasts into a second-level full factorial model with the within-subject factor “trial part” and the between-subject factor “substance”. We then used this model to obtain effects of ketamine on regions sensitive to the social task. We thus tested the following eight contrasts: (1) ketamine < placebo, trial part (TP): question; (2) ketamine > placebo, TP: question; (3) ketamine < placebo, TP: video; (4) ketamine > placebo, TP: video; (5) ketamine < placebo, TP: response; (6) ketamine > placebo, TP: response; (7) ketamine < placebo, TP: confidence rating; (8) ketamine > placebo, TP: confidence rating.

Parameter estimates from a region of interest (ROI) in the right pSTS region, identified in one of the t-tests (see Results), were exported using MarsBaR in SPM12^45^.

Effects of ketamine on functional connectivity between the pSTS ROI and other areas of the brain were assessed using a generalized psychophysiological interactions (gPPI) analysis with the CONN-toolbox in SPM 12^46^. For each participant, eigenvariates of the BOLD signal in the pSTS ROI were extracted and used to create a set of psychophysiological interaction (PPI) factors, which are an interaction of the deconvolved pSTS BOLD signal^47^ and the psychological factors of interest, namely the “question” part of the social and physical trials. The gPPI toolbox then inserted these PPI regressors into the individual GLMs described above and fitted these new GLMs to the BOLD data to yield functional connectivity parameter estimate maps.

Contrast images between functional connectivity during the social and physical question part of the trial (SOC > PHY) were then separately assessed in the participants of the placebo and the ketamine group using random effects one-sample t-tests in SPM 12; the effect of ketamine was assessed by comparing these contrast maps across groups using an independent samples t-test. All results of the fMRI analyses were considered significant if they exceeded the threshold of p < 0.05 after family-wise error correction for multiple comparisons at the cluster level across all the voxels of the brain, based on an uncorrected threshold (= cluster-forming threshold) of p < 0.001 at the voxel level. These results are indicated as p_(corr)_ in the text.

### Analysis of the behavioural data

Behavioural data were analyzed using SPSS 27 (IBM Corp., Armonk, USA). Effects of substance on PANSS were tested using independent samples *t*-tests for each of the five PANSS factors. Responses in the modified MASC were assessed in two ways: (1) in terms of accuracy (% correct responses in the social and physical trials) using a two-way, mixed analysis of variance (ANOVA) (between-subjects factor: placebo vs. ketamine ; within-subject factor: social vs. physical); and (2) subsequently, only for the social trials, by comparing the number of times each type of response (mentalizing, over-mentalizing, under-mentalizing, no mentalizing) was given by participants of the two groups using a one-factorial ANOVA.

## Results

### Effects of ketamine on symptoms of schizophrenia

Participants under ketamine showed significantly more schizophrenia-related symptoms than controls in four of the five factors used to assess the PANSS data: positive symptoms (*t*(33.52) = − 5.42, *p* < 0.001, *d* = − 1.46), cognitive disorganization (*t*(51.57) =  − 4.21, *p* < 0.001, *d* = − 1.12), negative symptoms (*t*(47.49) =  − 3.74, *p* < 0.001, *d* = − 1.05) and depression/anxiety symptoms (*t*(33.26) =  − 3.48, *p* = 0.001, *d* = − 0.94); no significant change in hostility symptoms was found (*t*(47,49) =  − 1.13, *p* = 0.264). In response to a reviewer’s suggestion, we calculated correlations between PANSS subscales and mentalizing performance in the ketamine group, but found no significant associations. The results are reported in Supplementary Table S1.

### Effects of ketamine on mentalizing

**Figure 2:**
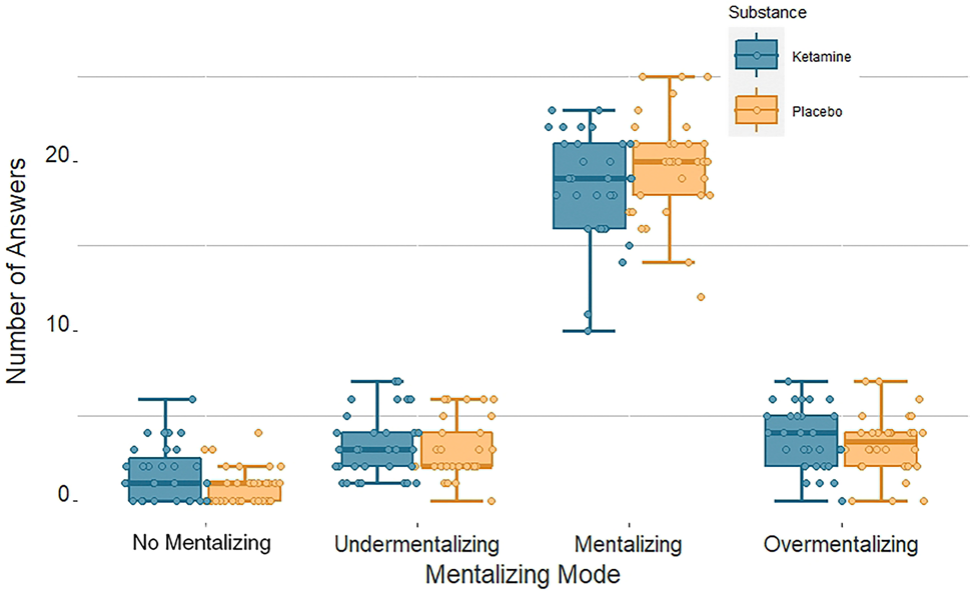
Effects of ketamine on mentalizing: participants under ketamine gave more “no mentalizing” answers in our movie-based theory-of-mind task (modified MASC) than controls; *MASC* movie for the assessment of social cognition.

Participants made more errors in the social than in the physical trials of the modified MASC task (*F(*1, 61) = 5.84, *p* = 0.019, ${\eta }^{2}$ = 0.087), and participants in the ketamine group made more errors than participants in the placebo group (*F*(1, 61) = 6.52, *p* = 0.013, ${\eta }^{2}$ = 0.097). The interaction between the factors trial type and participant group was not significant (*F*(1, 61) = 0.93, *p* = 0.34). The physical trials served only as control condition for the assessment of the BOLD response during social trials, in which participants were engaged in mentalizing, the main focus of our experiment. To assess if ketamine influenced the level of mentalizing that participants engaged in, we compared the frequency with which participants of both groups chose each possible type of answer. We found that ketamine affected the pattern of responses (Fig. 2; one-way ANOVA: “no mentalizing”: *F*(1,61) = 4.78, *p* = 0.033, ${\eta }^{2}$ = 0.073; “undermentalizing”: *F*(1,61) = 0.18, *p* = 0.672, ${\eta }^{2}$ = 0.003; “mentalizing”: *F*(1,61) = 2.45, *p* = 0.123, ${\eta }^{2}$ = 0.039; “overmentalizing”: *F*(1,61) = 0.45, *p* = 0.505, ${\eta }^{2}$ = 0.007). Thus, ketamine led to an increase in “no mentalizing” responses.

### Neural correlates of mentalizing under ketamine

**Figure 3:**
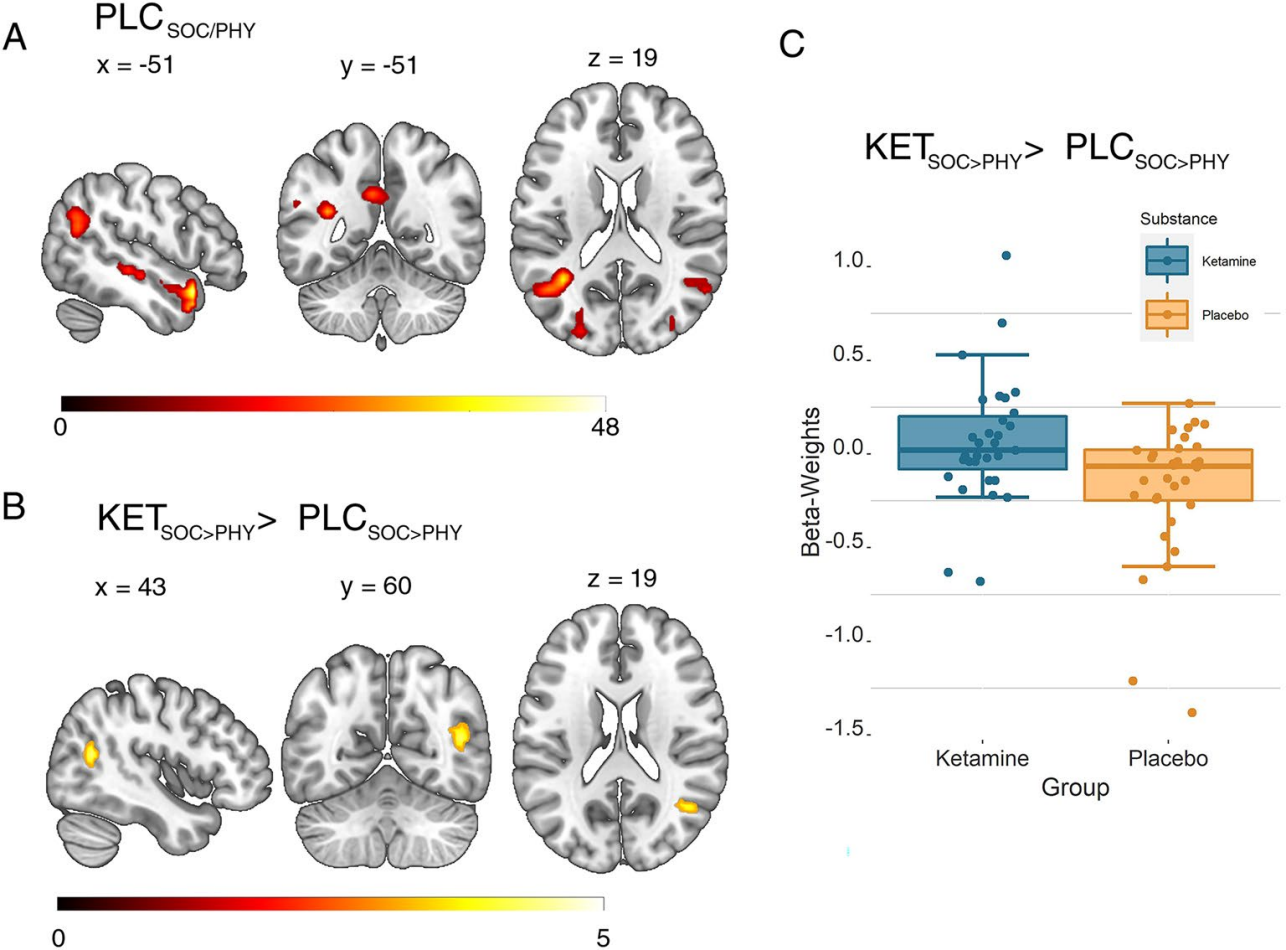
Neural correlates of social cognition and effects of ketamine during the modified MASC task. Threshold at p(FWE-cluster level) = 0.05, based on a voxelwise uncorrected threshold of p < 0.001. (**A**) Activation changes in left pSTS and precuneus in the placebo participant group during the question phase of the trial. (**B**) Increased activation in the right pSTS during social cognition under ketamine in the question condition (independent-samples t-test comparing the social > physical contrast images across participant groups). (**C**) Contrast of parameter estimates in the right pSTS cluster depicted in (**B**). *BOLD* blood-oxygen-level-dependent, *FEW* correction for family-wise errors, *KET* ketamine, *MASC* movie for the assessment of social cognition, *PLC* placebo, *PHY* physical, *SOC* social, *STS* superior temporal sulcus.

We expected social trials to evoke BOLD signal increases in social brain regions when compared to physical trials (control). This manipulation check was performed on the data of the placebo participant group creating a second-level model, using the SOC > PHY contrast images for the question part of the trial. Results (Fig. 3A) revealed changes in activation in the social compared to the physical trials in left pSTS [MNI-coordinates of activation peak: x = − 48, y = 17, z = − 19, ${k}_{e}$ = 193, *F*(1,244) = 47.66, p_(Corr)_ < 0.001; x = − 36, y = − 55, z = 20, ${k}_{e}$ = 175, *F*(1,244) = 38.02, p_(Corr)_ < 0.001], cuneus [x = − 21, y = − 97, z = 5, ${k}_{e}$ = 317, *F*(2,244) = 33.34, p_(Corr)_ < 0.001], middle occipital gyrus [x = 30, y = − 94, z = 11, ${k}_{e}$ = 222, *F*(1,244) = 31.63, p_(Corr)_ = 0.019], precuneus [x = − 6, y = − 52, z = 32, ${k}_{e}$ = 47, *F*(1,244) = 25.72, p_(Corr)_ = 0.019] and right pSTS [x = 54, y = − 61, z = 20, ${k}_{e}$ = 36, *F*(1,244) = 25.72, p_(Corr)_ = 0.054]. This pattern of findings is compatible with involvement of brain regions associated with social cognition during the social trials^48^.

We proceeded to evaluate effects of ketamine on social cognition during the MASC by comparing, for each of the four trial parts, the social with the physical trials (SOC > PHY) between groups. We found a significant increase in BOLD signal in the ketamine group during presentation of the questions in a cluster located in the right posterior superior temporal sulcus region [pSTS; x = 45, y = − 58, z = 17, ${k}_{e}$ = 52, *t*(244) = 4.42, *p*_(Corr)_ = 0.02; Fig. 3B,C]. No other differences between participant groups were found. The BOLD-signal from this pSTS-cluster was used as seed for a functional connectivity analysis (see next section).

**Figure 4:**
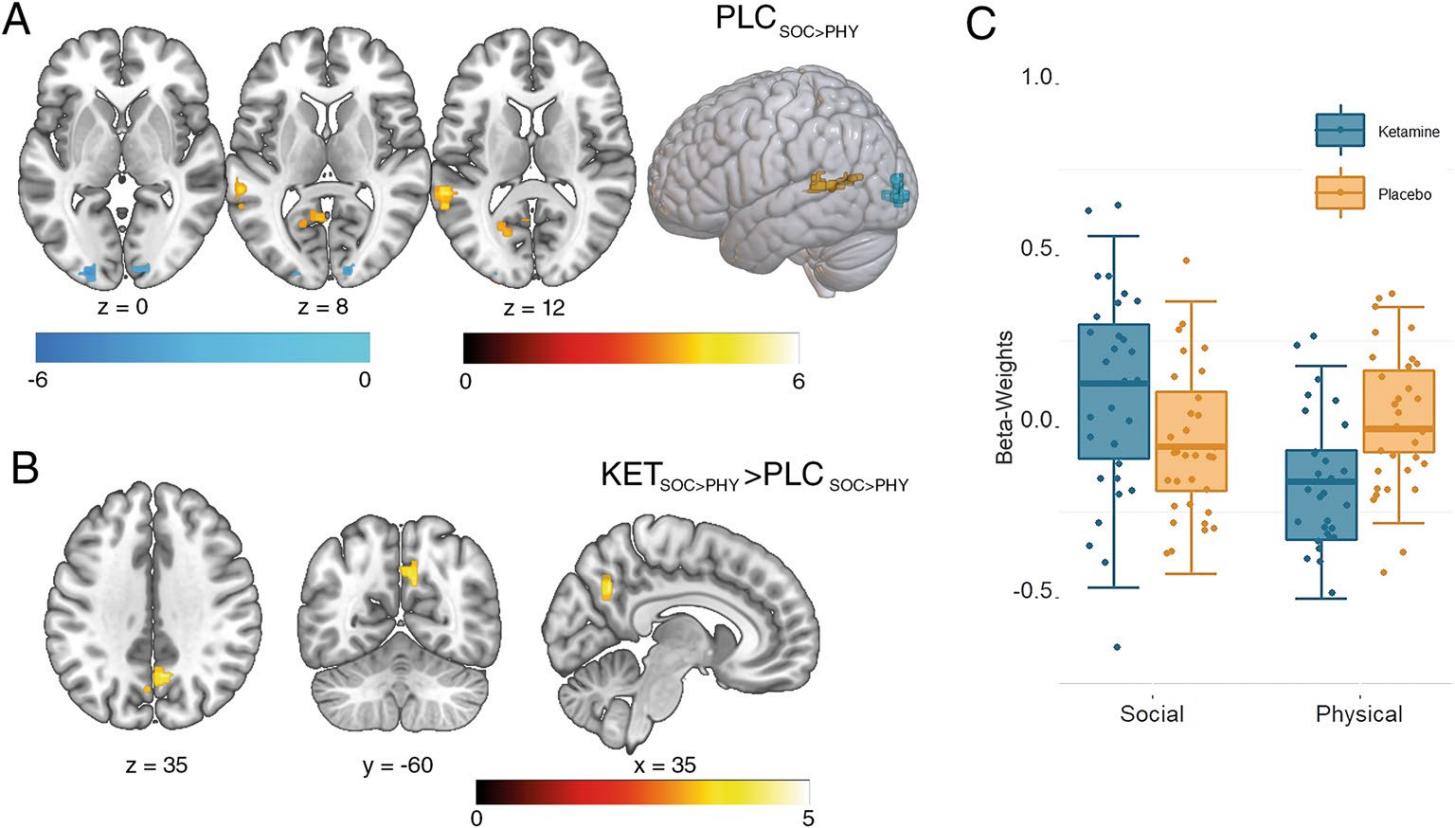
Functional connectivity during social cognition and effects of ketamine. Threshold at p(FWE-cluster level) = 0.05, based on a voxelwise uncorrected threshold of p < 0.001. (**A**) Under placebo, several regions showed changes in functional connectivity with the right pSTS cluster (Fig. 3B) during the question phase of the social compared to the physical trials: connectivity with STG and posterior cingulate increased, while connectivity with the cuneus and middle occipital gyrus decreased. (**B**) Under ketamine, one cluster in the anterior precuneus showed a larger difference in connectivity with the right pSTS cluster between social and physical trials. (**C**) Functional connectivity beta-weights in the cluster shown in (**B**). *KET* ketamine, *MTG* middle temporal gyrus, *PHY* physical, *PLC* placebo, *SOC* social.

Clusters of voxels that exhibited increased functional connectivity with the right pSTS in the placebo group during the question phase of the social compared to the physical trials were found (see Fig. 4A, warm colours) in left superior temporal gyrus [x = − 56, y = − 40, z = 10, ${k}_{e}$ = 162, *t*(31) = 5.80, *p*_(Corr)_ < 0.001] and posterior cingulate gyrus [x = 0, y = − 62, z = 4, ${k}_{e}$ = 110, *t*(31) = 5.22, *p*_(Corr)_ = 0.003]. Decreases in connectivity with right pSTS during the social compared to the physical trials were found (see Fig. 4A, cold colours) in cuneus [x = 16, y = − 92, z = 4 ${k}_{e}$ = 122, *t*(31) =  − 5.14, p_(Corr)_ = 0.002] as well as in middle occipital gyrus [MNI peak coordinates: x, y, z: − 24, − 94, − 2, ${k}_{e}$ = 111, *t*(31) = − 4.97, *p*_(Corr)_ = 0.003]. In the ketamine group, clusters of voxels showing higher connectivity with the right pSTS during the question phase of the social compared to the physical trials were found in anterior precuneus (x = − 2, y = − 68, z = 28, ${k}_{e}$ = 212, *t*(30) = 4.98, *p*_(Corr)_ < 0.001) and left middle temporal gyrus [x = − 50, y = − 46, z = 4, ${k}_{e}$ = 164, *t*(30) = 5.92, *p*_(Corr)_ < 0.001; x = − 56, y = − 10, z = − 4, ${k}_{e}$ = 127, *t*(30) = 5.87, *p*_(Corr)_ = 0.001]; clusters showing lower connectivity with the right pSTS in the social compared to the physical trials were identified in posterior precuneus [x = 24, y = − 76, z = 40, ${k}_{e}$ = 244, *t*(30) =  − 5.81, *p*_(Corr)_ < 0.001] and superior occipital gyrus [x = 32, y = − 86, z = 22, ${k}_{e}$ = 107, *t*(30) =  − 6.51, *p*_(Corr)_ = 0.003]. Of particular importance for our study, a t-test for independent samples across participant groups revealed one cluster showing a larger difference in connectivity with the right pSTS between social and physical trials under ketamine compared to placebo, in the anterior precuneus [see Fig. 4C; x = 6, y = -58, z = 34, ${k}_{e}$ = 130, *t*(61) = 4.77, *p*_(Corr)_ = 0.042].

## Discussion

The present study indicates that a dose of ketamine inducing positive and negative symptoms associated with schizophrenia impairs mentalizing during the observation of social interactions and increases neural activity in right pSTS as well as connectivity between right pSTS and anterior precuneus during this task. These findings suggest that intravenous subanesthetic ketamine impairs social cognition and affects its neural correlates, supporting a link between a proposed psychopathological mechanism in schizophrenia and deficits in social cognition associated with that disorder. However, it should be mentioned that these deficits were not limited to social cognition, as participants were also presenting with reduced neurocognitive functioning in the control-condition.

The MASC task used here allows to characterize mentalizing deficits specifically by differentiating over- from undermentalizing^34^. It has revealed undermentalizing in individuals with schizophrenia and their unaffected relatives^8,35^, as well as in individuals with autism spectrum disorder (ASD)^49^. In the present study, participants under ketamine also mentalized less (i.e., gave significantly more “no mentalizing” answers). These findings lead to the question of how NMDA-receptor blockade by an agent like ketamine might cause a reduction in mentalizing, and whether such mechanisms might be occurring in schizophrenia or ASD. As previously mentioned, changes in glutamate signaling lead to dysfunctions in social cognition in animal models, in particular to a disruption of social preference (shown by relative preference for social over non-social stimuli)^24^, a key factor for the development of healthy social cognition that is deficient in schizophrenia^50^.

While no study has investigated the causal effects of acute blockade of NMDA-receptors on mentalizing in healthy volunteers, several correlational studies have linked high glutamate concentration, or high glutamate to GABA ratios, with social cognitive dysfunction. For example, glutamate concentration in dorsolateral prefrontal cortex of healthy participants measured with magnetic resonance spectroscopy (MRS) was negatively correlated with perspective taking scores in a task designed to assess empathy in people with schizophrenia and ASD^51^. Another MRS study revealed that healthy people with higher scores in an ASD/schizotypy-questionnaire, which quantifies social dysfunction, exhibit a higher glutamate concentration in the superior temporal region (ST) bilaterally and increased glutamate to GABA ratio in the right ST^52^. Links between increased glutamate and dysfunctions of mentalizing have also been reported in schizophrenia and ASD^1,11,53^. For example, a negative correlation between total glutamatergic metabolites in the left thalamus and social functioning has been found in people with schizophrenia^54^. In ASD, GABA receptor down-regulation is thought to lead to cortical disinhibition, which also leads to an increased excitation/inhibition ratio^52^. One study reported that higher glutamate levels in the right superior temporal cortex were related to poorer social and interpersonal skills in people with ASD, and that this relationship was increasingly strong when GABA was reduced^55^. Overall, there seems to be a functional link between altered glutamate signaling and complex social cognition in ASD and schizophrenia, the underlying mechanisms of which are not yet fully understood^56^.

Our results support this hypothesis by demonstrating causal alteration of mentalizing in participants undergoing NMDA-receptor blockade by ketamine. We note that we cannot make conclusions specific to mentalizing, as performance in the non-social condition of our task was also reduced under ketamine. The lower performance observed in both the control and social cognition conditions within the ketamine group can possibly be partly attributed to the dissociative effects of ketamine^57^. Additionally, we cannot rule out the possibility that stress might be a mediator driving changes in behavior. A study found that ketamine evoked stress-like alertness in healthy participants^58^ correlating with hyperconnectivity of hippocampus and precuneus at rest. As such, an alternative explanation to our findings might be that participants experience stress as result of changes to their perception, which in turn leads to a well-established, stress-induced decrease of memory function, resulting in worse performance in a variety of tasks. This possibility should also be addressed in future studies when looking at effects of ketamine on task performance.

At the neural level, we found activation in left pSTS, cuneus, middle occipital gyrus, precuneus and right pSTS during the question phase of the MASC in the placebo group during our manipulation check. Some of these regions (pSTS and precuneus) are part of the mentalizing network and are thus typical of activations expected in a social cognition task^14^. However, activations in cuneus and middle occipital gyrus are not typically linked to the mentalizing network, and may reflect the complexity of the task structure, in which multi-part trials build sequentially on each other in the social condition. Ketamine increased differences in the right posterior STS BOLD signal evoked by answering a question related to emotions or intentions of others (mentalizing condition), compared to a non-social control question. Activation in the pSTS, particularly in the right hemisphere, is frequently observed during mentalizing^59^. For instance, a meta-analysis by Schurz and colleagues revealed that mentalizing evoked by different sets of social animation tasks was associated with a high probability of activation in the right pSTS^60^. Our results are also compatible with findings in participants with schizophrenia: for example, one study using comic strips reported increased activity during mentalizing compared to a non-social control task in a very closely located pSTS-cluster^13^, while a different study using static face stimuli reported higher activation in another right pSTS location during mentalizing compared to emotion recognition in patients compared to controls^16^. Repeatedly observed hyperactivity of pSTS in schizophrenia during social tasks led the authors to state increased BOLD activity in pSTS might constitute an endophenotype of schizophrenia^61^. Severity of positive symptoms was positively correlated to observed disinhibition of pSTS during social cognition in another recent study^62^. The authors used dynamic causal modeling to estimate connectivity between two brain regions active during motion perception (both, social and non-social) and found that connectivity increased between V5 and pSTS in patients, concluding that this increased connectivity might contribute to wrongful attribution of social states to agents.

Several regions showed changes in functional connectivity with the seed cluster under placebo during the question phase (social > physical): connectivity with STG and posterior cingulate increased, possibly reflecting reasoning about mental states of others and oneself^63,64^. The fact that, compared to other studies, we found decreased connectivity with cuneus and middle occipital gyrus could potentially be attributed to the complexity of the naturalistic mentalizing task performed by our participants or the use of PPI to investigate functional connectivity in our study. Decreased connectivity between these regions might reflect the participants’ attempt to focus more on social situations and less on objects^65^. When compared to ketamine, one cluster in the anterior precuneus showed a larger difference in connectivity between the social compared to the physical condition under ketamine. Generally, activation in anterior precuneus is frequently observed during mentalizing^66^ and self-related consciousness in healthy participants^67^. One study in healthy participants reported increased task-based connectivity in the (“mentalizing”-)network of the dmPFC, pSTS and precuneus during a task evoking spontaneous mentalizing^68^. The authors concluded that the integrative functions of the precuneus, along with its association with self-awareness might explain why social cognition tasks lead to an increase in connectivity between these regions. Findings from two studies in patients with schizophrenia support this idea: increased perfusion of the precuneus, measured with single photon emission computed tomography, was correlated to better insight into one’s own mental disorder^67^, while functional connectivity of precuneus negatively correlated with apathy in another study^69^.

Coactivation of pSTS and precuneus in healthy participants has been observed during naturalistic social tasks involving reasoning about emotional and mental states of others^66,70^. However, also more abstract tasks that involve animacy and biological motion have been linked to connectivity between right pSTS and precuneus in healthy participants^71^. This is unsurprising, as both precuneus and pSTS play an important role in the mentalizing network. Increased functional connectivity between pSTS and precuneus in patients with schizophrenia compared to controls was found during processing of ambiguous word pairs that could be understood either literally (e.g., “birth weight”) or as part of a metaphor (e.g. “sealed lips”)^72^. These connectivity findings may be related to abnormal glutamate and GABA signalling in schizophrenia, as these neurotransmitters are known to modulate functional connectivity in the healthy brain^73^. What might our functional connectivity findings represent? The authors of the above-mentioned study proposed that increased precuneus-pSTS connectivity reflects their patients’ attempt to compensate for their impaired metaphor comprehension^72^. Thus, both in schizophrenia and in our ketamine study, the increases in precuneus-pSTS functional connectivity may represent neural correlates of an attempt to compensate for abnormal neurotransmission. In this context, functional connectivity with precuneus might reflect a shift of attention, as observed e.g. during attentional deployment for emotion regulation when the attentional focus is moved away from negative emotional stimuli such as a car accident and towards something else. One study reported increased precuneus-to-amygdala functional connectivity when participants could freely view unpleasant images and focused their gaze on a non-arousing region^74^. Following this idea, we could propose that our connectivity finding represents a dysfunctional shift of attention away from a mentalizing perspective (needed to correctly identify intentions or emotions of others) and towards a more concretistic layer of attention (focused on superficial details of a situation). For patients with schizophrenia, this finding might imply that they miss social cues due to their heightened attention towards non-social attributes of their surrounding environment. This in turn might lead to difficulties with social interactions and thus, finally, to social avoidance, leading to diminished social functioning^3^. The scarcity of pSTS-related connectivity findings in schizophrenia, however, constrains our interpretation attempts and calls for additional research.

### Limitations

Limitations in our study concern the behavioral task, the ketamine-model for psychosis and whether changes evoked by ketamine reflect changes in glutamate-signalling. In our task, participants relied both on visual and acoustic features of the video clips to perform the social task, while solely visual information mattered in the physical condition, which yields asymmetry between the conditions.

A limitation of the ketamine-model for psychosis comes from the smaller impact on PANSS levels of ketamine (mean of 40 in our and similar studies^41,75^) compared to the PANSS levels observed during clinically relevant psychotic symptoms in schizophrenia (values of > 75 are frequently observed, e.g. Leucht et al.^76^).Therefore, comparisons between the effect of our manipulation and the effect of psychosis in schizophrenia on mentalizing should be made with caution. In addition, blinding is difficult in ketamine studies^77^, partly due to nausea and disorientation at the start of the ketamine infusion^57^. To minimize these effects, we only recruited ketamine-naïve participants who were unlikely to be able to tell whether their experience was related to ketamine or to undergoing MRI. As using a control substance such as midazolam might result in unwanted influences of neural activation^78^, we decided against this strategy. Conclusively, blinding in our study may have been single- rather than double-blind, in particular for the ketamine-receiving group.

Finally, since we did not measure glutamate levels after administering ketamine, conclusions about whether the fMRI results were caused by changes in glutamate transmission cannot be drawn. Even though most MRS studies showed that ketamine causes changes in glutamatergic transmission in anterior cingulate, not all studies were able to replicate these effects^75,79,80^. An early study reported that the direction of these changes in the prefrontal cortex varied as a function of the amount of ketamine administered^81^. Unfortunately, as of today, no studies have explored direct manipulation of the glutamatergic system within our regions of interest, i.e., pSTS or precuneus. Future studies could therefore explore the direction of change in glutamatergic transmission following administration of subanesthetic doses of ketamine in mentalizing regions.

Taken together, our findings show that people who received ketamine were exhibiting neurocognitive and social cognitive deficits in a naturalistic mentalizing-task. They mentalized less and demonstrated changes in BOLD-activity and task-related connectivity in right pSTS and precuneus, hinting to how glutamatergic neurotransmission might be tied to emergence of these deficits in disorders like schizophrenia.

### Supplementary Information

Supplementary Information.
