# Supplementary material for: Effects of NMDA-receptor blockade by ketamine on mentalizing and its neural correlates in humans: a randomized control trial
Source: Sci Rep. 2023 Oct 11;13:17184. doi: 10.1038/s41598-023-44443-6 (PMC10567921; doi:10.1038/s41598-023-44443-6)
Supplement: Supplementary file 1 — Supplementary Information. [file 41598_2023_44443_MOESM1_ESM.docx]

# Supplemental Material

# *Title: Effects of NMDA-receptor blockade by ketamine on mentalizing and its neural correlates in humans – a randomized control trial*

Sven Wasserthal (1), Mirko Lehmann (2), Claudia Neumann (3), Achilles Delis (3), Alexandra Philipsen (4), René Hurlemann (5), Ulrich Ettinger (2), Johannes Schultz (6,7)

(1) Division of Medical Psychology, Department of Psychiatry and Psychotherapy, University Hospital of Bonn, Bonn, Germany

(2) Department of Psychology, University of Bonn, Bonn, Germany

(3) Department of Anaesthesiology and Intensive Care Medicine, University Hospital Bonn

(4) Department of Psychiatry and Psychotherapy, University Hospital of Bonn, Bonn, Germany

(5) Department of Psychiatry, School of Medicine & Health Sciences, University of Oldenburg, Oldenburg, Germany

(6) Center for Economics and Neuroscience, University of Bonn, Bonn, Germany

(7) Institute for Experimental Epileptology and Cognition Research, Medical Faculty, University of Bonn, Bonn, Germany

## Adapted MASC:

A detailed description of the original procedure can be found in Dziobek et al. [1]. In the present experiment, we adapted this task in the following two ways: (1) we split up scenes longer than 30 seconds into shorter clips to avoid evoking task-related BOLD responses in frequency bands in which low-frequency MRI signal noise is commonly found (<https://imaging.mrc-cbu.cam.ac.uk/imaging/DesignEfficiency#Signal-processing>); and (2) we added non-social control questions about the physical surroundings of the actors in the video to allow contrasting BOLD signal evoked by social and non-social cognition (e.g., “What was the color of the curtain?”). In our version of this experiment, there were 54 scenes and 27 social and physical questions. The social questions were selected from the original MASC set [1,2]. The physical questions were created by us and inserted into the 11 newly created splits between scenes; the remaining 16 were used to replace randomly selected social questions. Video clips lasted about 10 seconds on average; the total duration of the video clips was 541 seconds; the order of scenes and questions was identical for all participants. Each of the 54 scenes constituted one trial, due to the comprehensive nature of the presented findings, confidence rating data are not further discussed in the present manuscript. Written instructions were given before entering the scanner and repeated onscreen in the scanner prior to MRI data acquisition. The task was presented and responses were collected using the software Presentation (Version 18.1, Build 02.01.15; NeuroBehavioral Systems Inc., Berkeley, CA, USA). Participants held “ResponseGrip Hardware” (NordicNeuroLab AS, Bergen, Norway) in each hand that allowed for a button press with either thumb or index finger. The index finger buttons were used to select an answer, pressing either thumb button locked the answer and triggered the next stimulus.


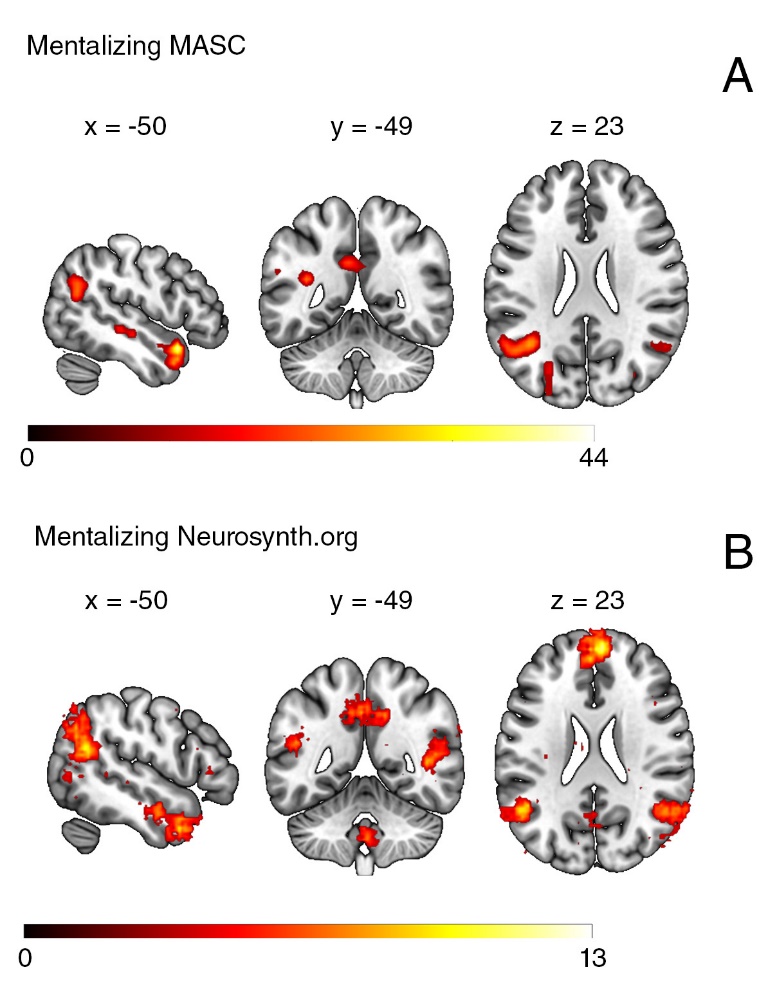


Figure 1: Comparison between activation (A) during the question phase of the MASC in participants of the placebo-group and (B) an activation map showing regions active during various mentalizing tasks obtained from neurosynth.org.

## Correlations PANSS x Mentalizing:

Table 1. Pearson-Correlations between single PANSS scales and mentalizing in the ketamine-group. Critical p-value was p(crit) = .01 after Bonferroni-correction

| Condition | r | p (corr.) |
| --- | --- | --- |
| Mentalizing x PANSS Positive | .157 | .426 |
| Mentalizing x PANSS Cognitive Disorganization | .140 | .477 |
| Mentalizing x PANSS Hostility | -.052 | .794 |
| Mentalizing x PANSS Negative | -.284 | .143 |
| Mentalizing x PANSS Depression Anxiety | .084 | .672 |

Since mentalizing is generally thought to be impacted by the severity of negative symptoms in schizophrenia, we added this brief analysis to show whether there is a significant correlation between these variables in our analysis. Table 1 shows that none of the PANSS dimensions correlate significantly (critical p-value corrected for multiple corrections p_(crit)_ = .01) with mentalizing. However, the strongest correlation within this analysis was between mentalizing and the PANSS negative scale (r = -.284), which is in accord with the literature. However, since we did not design our study to analyze multiple correlations, these results should be viewed with caution.

References:

[1] Dziobek I, Fleck S, Kalbe E, Rogers K, Hassenstab J, Brand M, et al. Introducing MASC: a movie for the assessment of social cognition. J Autism Dev Disord 2006;36(5):623–36.

[2] Montag C, Dziobek I, Richter IS, Neuhaus K, Lehmann A, Sylla R, et al. Different aspects of theory of mind in paranoid schizophrenia: evidence from a video-based assessment. Psychiatry Res 2011;186(2-3):203–9.
